# Exploring Linalool‐Based Phytotherapy for Excitatory/Inhibitory Imbalance in Alzheimer's Disease: A Review of Lavender and Cannabis Therapeutic Effects on Sleep, Seizures, and Cognition

**DOI:** 10.1002/ptr.70191

**Published:** 2026-02-24

**Authors:** Ilaria Piccialli, Giovanni Roviello, Giorgia Magliocca, Emilia Esposito, Anna Pannaccione

**Affiliations:** ^1^ Division of Pharmacology, Department of Neuroscience, Reproductive and Dentistry Sciences School of Medicine, University of Naples Federico II Naples Italy; ^2^ Institute of Biostructures and Bioimaging, Italian National Council for Research (IBB‐CNR) Naples Italy

**Keywords:** Alzheimer's disease, cannabis, entourage effect, excitatory/inhibitory imbalance, lavender, linalool

## Abstract

Alzheimer's disease (AD) is a progressive neurodegenerative disorder characterized by cognitive decline, memory impairment, and behavioral disturbances. While its pathogenesis is complex, increasing evidence supports the role of an imbalance between excitatory and inhibitory (E/I) neuronal activity in disease progression. E/I imbalance contributes to synaptic impairment, network hypersynchrony, and ultimately cognitive deterioration. Despite advances in understanding AD pathophysiology, no highly effective or disease‐modifying treatments are currently available. This review explores the pharmacological properties of linalool, a monoterpene found in high concentrations in *Lavandula angustifolia* and in *Cannabis sativa* L. as minor component, which has demonstrated several neuromodulatory effects. Unlike current AD therapies that typically target cholinergic or NMDA‐mediated mechanisms in isolation, linalool exerts a multi‐target action that may help restore E/I balance. These effects also underlie its well‐established anxiolytic and sedative properties, though their therapeutic relevance extends beyond behavioral symptoms to include the modulation of neuronal network function in neurodegeneration. We also examine studies on lavender extracts, rich in linalool, with the potential to influence sleep regulation, neuronal excitability, and cognitive function. Finally, we discuss the emerging role of cannabis extracts and the potential entourage effect of linalool and phytocannabinoids in targeting neuronal excitability. Overall, the findings discussed in the present review highlight linalool‐containing phytocomplexes as promising candidates for supportive or complementary strategies in managing E/I imbalance and cognitive decline in AD.

## Graphical abstract

Schematic overview of the proposed neuromodulatory actions of linalool in Alzheimer's disease. Through multi‐target effects on oxidative stress, amyloid aggregation, GABAergic and glutamatergic signaling, linalool may restore excitatory/inhibitory balance. These mechanisms potentially underlie beneficial effects on neuronal hyperexcitability, sleep disturbances, seizure susceptibility, and cognitive impairment.

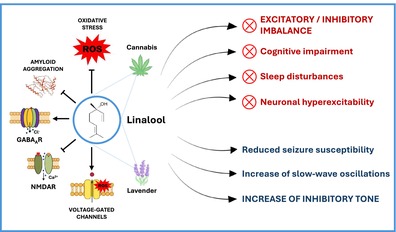

## 1 Introduction

Alzheimer's disease (AD) is the most common form of dementia in the elderly, accounting for up to 80% of all cases, either as a primary pathology or in combination with other conditions contributing to dementia, such as cerebrovascular diseases. A progressive and unrestrainable deterioration of cognitive function is the main pathological feature of AD while typical histopathological markers are the accumulation of the Amyloid β (Aβ) protein as diffuse and neuritic plaques and the abnormal phosphorylation and aggregation of the protein Tau, which results in neurofibrillary tangles (Selkoe 1991). Although an enormous amount of evidence points to Aβ and Tau as major culprits in the AD pathogenesis, treatments targeting these proteins, including those recently approved (Kim et al. 2025), have thus far displayed poor or no effectiveness in modifying the disease course, while the available therapies targeting cholinergic and glutamatergic mechanisms only partially counteract the cognitive symptomatology (Ibrahim et al. 2020).

A large body of evidence has accumulated suggesting the substantial pathogenic relevance of excitatory/inhibitory (E/I) imbalance in AD (Maestú et al. 2021). Neuronal hyperexcitability along with the correlated network abnormalities may indeed disrupt fundamental brain communication mechanisms and interfere with proper information processing, hence leading to cognitive impairment and contributing to the spreading of the amyloid pathology (Palop and Mucke 2009, 2010; Wu et al. 2016). At the cellular level, aberrant neuronal excitability may promote the abnormal production and release of amyloid species into the interstitial fluid, as a result of increased neuronal firing and consequent high metabolic demand, induce neuronal damage, and foster neurodegeneration (Cirrito et al. 2008). At the network level, neuronal hyperactivity (1) affects gamma oscillations, which are fundamental for cognitive function, (2) induces cortical and hippocampal hypersynchrony—a pattern of abnormally synchronized neuronal firing—which causes both seizures and the progressive degradation of memory processes, and (3) interferes with the physiological slow‐wave activity, causing sleep disturbances and subsequent disruption of sleep‐dependent memory consolidation (Styr and Slutsky 2018; Vossel et al. 2013; Katsuki et al. 2022; Horvath et al. 2021).

Notably, neuronal hyperexcitability can be present in AD in its most severe form, epilepsy. Since the earliest cases reported by Alois Alzheimer, it has been well known that AD patients, especially those with early‐onset forms, have an augmented risk of developing spontaneous seizures and epilepsy in comparison to healthy age‐matched controls (Leo et al. 2022; Vossel et al. 2017; Helmstaedter and Witt 2017). Besides a high seizure risk, an increased prevalence of subclinical epileptiform activity has also been described in patients with clinically diagnosed AD (Lam and Noebels 2020; Vossel et al. 2016). Such subclinical epileptiform activity remains largely under‐recognized, although its impact on the clinical course of AD can significantly influence the quality of life of patients and their families and worsen the socioeconomic burden of AD.

In this context, E/I imbalance‐targeting strategies have emerged as a potential therapeutic approach to modify the AD progression. Results obtained in AD animal models suggest the effectiveness of anti‐seizure drugs in ameliorating synaptic dysfunction and cognitive deficits (Sanchez et al. 2012). Similarly, the reduction of hippocampal hyperactivity with low doses of levetiracetam was shown to improve cognition in mild cognitive impairment patients (Bakker et al. 2012). However, the overall impact of these strategies on cognitive functions remains uncertain, as clinical trials in AD patients have yielded mixed results (Kamondi et al. 2024). Although classical antiseizure medications such as carbamazepine or valproate have shown limited tolerability and suboptimal efficacy in AD, even newer agents like brivaracetam or lamotrigine have failed to provide targeted modulation of the specific excitability alterations underlying AD pathology (Leo et al. 2022).

These observations further reinforce the idea that targeting E/I imbalance through isolated mechanisms may not be sufficient to restore network homeostasis in AD. In this regard, there is growing recognition that the dominant pharmacological paradigm, which remains focused on single‐target approaches, does not align with the multifactorial nature of AD. This also applies to the approaches aimed at counteracting E/I imbalance. In fact, despite the increasingly recognized relevance of excitability‐related dysfunction in AD, the single‐pathway interventions tested thus far have proven ineffective in restoring network homeostasis while preserving cognitive function, reflecting the broader limitation of a model that overlooks the interdependence of distinct regulatory pathways within the neuronal network. A shift toward polypharmacological approaches, conceived as the rational modulation of interconnected pathophysiological nodes, such as synaptic transmission, neuronal excitability, redox balance, and neuroimmune signaling, could offer a more appropriate framework. Within this perspective, multi‐target compounds capable of modulating both inhibitory and excitatory transmission in a homeostatic fashion, possibly coupled with anti‐inflammatory and antioxidant properties, may represent a promising direction for future pharmacological interventions.

In light of this, medicinal plants are increasingly viewed as valuable sources of multi‐target agents capable of interfering with the complex pathophysiology of AD. Notably, a recent taxonomic analysis of botanicals traditionally used in the management of cognitive disorders identified several families, most prominently Lamiaceae, Fabaceae, and Apiaceae, associated with neuroactive phytochemicals relevant to AD (Kamran et al. 2020). These families include species rich in monoterpenes, flavonoids, and alkaloids, with documented activity on neurotransmission, redox balance, and neuroinflammation, key processes involved in AD‐related network dysfunction. Several phytochemicals have already shown efficacy in alleviating behavioral and psychiatric symptoms associated with AD (Lin et al. 2007; Scuteri et al. 2017; Perry and Perry 2006), while others are currently being investigated for their effects on neuronal excitability. Despite the long‐standing recognition of anticonvulsant properties in various medicinal plants, this knowledge has had limited impact on mainstream therapeutic strategies, partly due to the lack of rigorous identification of active constituents. Nonetheless, some plant‐derived compounds have recently undergone development as antiepileptic drugs, including cannabidiol (CBD) and cannabidivarin from *Cannabis sativa* L., and huperzine A from *Huperzia serrata* (Thunb.) Trevis. (Challal et al. 2023).

Among the phytochemical classes involved in such effects, monoterpenes are particularly promising due to their broad neuropharmacological profile, including antioxidant, anti‐inflammatory, and neuromodulatory properties. Linalool, a major monoterpene component of lavender essential oil (EO) and a minor constituent of cannabis, has emerged as a candidate of interest for its dual action on glutamatergic and GABAergic neurotransmission, both of which are critically involved in the regulation of E/I balance. This dual action, along with the capability of influencing other pathophysiological mechanisms relevant to AD, including oxidative stress and neuroinflammation, places linalool among those compounds that deserve further investigation for their potential to modulate multiple, interconnected targets within dysfunctional neuronal networks.

This review hence aims to critically explore the neuropharmacological potential of linalool in the context of AD‐related network dysfunction, with particular focus on its relevance for the modulation of E/I imbalance. We will also review current evidence on the effects of *Lavandula angustifolia* Mill. and *Cannabis sativa* L. extracts on cognition, sleep regulation, and seizure susceptibility and discuss their potential relevance in managing cognitive decline and excitability‐related symptoms in AD, with particular attention to linalool's contribution as a bioactive component in complex formulations, such as those derived from cannabis, where its presence—despite being at low concentrations—may significantly influence the anticonvulsant activity of major phytocannabinoids.

## 2 Mechanisms Underlying Excitatory/Inhibitory Imbalance in Alzheimer's Disease

The imbalance between excitatory and inhibitory neuronal activities, in favor of excitatory, occurring in AD is a multifactorial phenomenon depending on the alterations of both synaptic inputs and intrinsic neuronal properties. Although several non‐amyloid‐induced alterations such as cholinergic deficits, inflammatory changes, and aberrant neurogenesis are likely involved in neuronal E/I imbalance, a key role has been attributed to Aβ and to its contribution to the development of neuronal hyperexcitability (Harris et al. 2020). Indeed, there is substantial mechanistic evidence that Aβ oligomers may directly induce neuronal hyperexcitability even before the manifestation of early AD symptoms (Brorson et al. 1995; Busche et al. 2008; Zott et al. 2019). In general, there are numerous demonstrations about a multifaceted correlation between amyloid pathology and E/I imbalance in AD, and the existence of a vicious cycle in which the Aβ pathology and neuronal hyperactivity drive each other may represent a key aspect of the AD progression to look at for shaping future pharmacological approaches.

The disruption of glutamate homeostasis and glutamatergic neurotransmission is a central aspect of E/I imbalance and has been proposed as a mechanism for the Aβ‐mediated neuronal hyperactivity. In in vitro and in vivo experimental models, Aβ was reported to increase hippocampal *N*‐methyl‐d‐aspartate (NMDA) receptor currents (Charkhkar et al. 2015; Alberdi et al. 2010; Molnár et al. 2004), and to block glutamate uptake by altering the expression and/or the function of glutamate transporters (Fernández‐Tomé et al. 2004; Matos et al. 2012), hence inducing glutamate spillover and excessive activation of extra‐ and peri‐synaptic glutamate receptors (Zott et al. 2019; Li et al. 2009). The aberrant activation of extra‐synaptic NMDA receptors, in turn, induces neuronal hyperexcitability and excitotoxic damage (Lerdkrai et al. 2018). Importantly, glutamate signaling abnormalities have been found in AD patients. In post‐mortem brain tissues from AD patients, a reduction in the levels of glutamine synthase was also observed and linked to an increase of glutamate availability (Le Prince et al. 1995).

The increase of the excitatory tone has also been linked to the effects of the Aβ aggregation and accumulation on the expression and function of several ion channels involved in membrane excitability and neuronal firing of excitatory neurons. For instance, functional alterations of the voltage‐gated Na^+^ (Na_V_) channel Na_V_1.6 induced by Aβ have emerged as a crucial factor in hippocampal neuron hyperactivity and Ca^2+^ dysregulation in AD experimental models (Ren et al. 2014; Ciccone et al. 2019; Piccialli, Ciccone, et al. 2021). In addition, several findings from different AD models have converged on the loss of the delayed rectifier K^+^ currents (*I*_DR_) mediated by the voltage‐gated K^+^ channel (K_V_) K_V_2.1, the main *I*_DR_ α subunit in pyramidal neurons, as a potential factor contributing to hippocampal neuron hyperexcitability (Frazzini et al. 2016; Wei et al. 2018; Piccialli, Sisalli et al. 2022).

Numerous studies have attributed a central role in E/I imbalance to the dysfunction of GABAergic neurotransmission (Ambrad Giovannetti and Fuhrmann 2019). The reduction of the inhibitor tone has been reported in different in vitro and in vivo models of AD and linked to the loss of GABAergic interneurons and to the decrease of both GABAergic signaling and responsiveness to GABAergic stimuli (Verret et al. 2012; Mao et al. 2024; Bie et al. 2022; Li et al. 2021). Importantly, several studies have demonstrated the detrimental role of Aβ aggregates in such GABAergic alterations (Krantic et al. 2012; Chung et al. 2020; Ulrich 2015). In addition, reduced GABAergic terminals have been observed on cortical neurons in proximity to Aβ plaques in both animal and human brains (Garcia‐Marin et al. 2009).

Parvalbumin‐expressing (PV+) neurons, a major class of GABAergic interneurons, play a key role in the maintenance of E/I balance (Campanac et al. 2013), and a huge body of evidence has been provided about their relevance in network oscillations and synchrony and, consequently, in learning and memory functions (Csicsvari et al. 2003; Ognjanovski et al. 2017). A significant decrease in the number of PV+ neurons has been found in several AD mouse models (Albuquerque et al. 2015; Takahashi et al. 2010) and in the brain of AD patients in those areas mainly affected by Aβ‐ and Tau‐pathology such as the entorhinal cortex and hippocampus (Fonseca et al. 1993; Mikkonen et al. 1999). In general, several defects in GABAergic transmission have been observed in AD patients and correlated with EEG disturbances (Chung et al. 2020; Soininen et al. 1988; Chen et al. 2021). Along with the reduction of PV+ neurons, the loss of somatostatin‐expressing (SST+) neurons has also been reported in AD patients, human post‐mortem AD brains, and mouse models of AD (Sanchez‐Mejias et al. 2020; Schmid et al. 2016). Of note, SST+ interneurons can strongly attenuate bursting of pyramidal cells in both the hippocampus and neocortex (Royer et al. 2012; Gentet et al. 2012). Moreover, they are involved in the generation of slow‐wave activity during the non‐rapid eye‐movement (REM) sleep, and their loss has been correlated to E/I imbalance and altered sleep brain oscillations in AD (Katsuki et al. 2022). Importantly, the selective activation of both SST+ and PV+ interneurons suppressed epileptiform activity in hippocampal slices (Ledri et al. 2014).

The hypofunction of GABAergic interneurons has been correlated to altered levels of the Na_V_1.1 channel, the Na_V_ channel subunit predominantly expressed by PV+ and SST+ neurons and required for their fast‐spiking properties (Ogiwara et al. 2007; Tai et al. 2014). A significant loss of Na_V_1.1 was observed in human AD brains and in multiple AD mouse models (Verret et al. 2012; Hamm et al. 2017) and has been correlated to reduced PV+ neurons' activity, increased network hypersynchrony, and epileptiform activity (Verret et al. 2012; Martinez‐Losa et al. 2018). Of note, the transplantation of Na_V_1.1‐overexpressing interneurons in AD mice counteracted network hypersynchrony and restored gamma oscillations (Martinez‐Losa et al. 2018), hence confirming that targeting inhibitory neurons could help rescue network alterations in AD.

As only partially described above, the mechanisms underlying E/I imbalance in AD involve a complex interplay of molecular and cellular processes. This highlights the need to further explore therapeutic strategies capable of addressing this intricate dysregulation, with multi‐target approaches potentially offering a valuable opportunity.

## 3 Linalool: Neuropharmacological Properties and Protective Effects in Preclinical Models of AD

Linalool is an acyclic monoterpene alcohol abundantly present in a series of aromatic plants, such as lavender, coriander, and bergamot, among others. Linalool, which exists in nature as two enantiomers, (R)‐(−)‐linalool and (S)‐(+)‐linalool, is also the biosynthetic precursor of several other alcohols and aldehydes including linalyl acetate, the second most abundant component after linalool in the EOs from many aromatic plants. Except for a linalool auto‐oxidation product, linalool hydroxide, which was reported to increase oxidative stress in vitro, linalool and its derivatives as well as the EOs rich in these compounds have been shown to be non‐toxic in several studies (Bickers et al. 2003; Smeriglio et al. 2018; López et al. 2017). Increasing evidence demonstrates that linalool can exert a variety of biological activities, such as anticancer, antimicrobial, and antifungal activities (Chhikara et al. 2018). Besides, several in vitro and in vivo studies have reported that linalool may provide protection for the central nervous system (CNS) by interfering with different key pathogenic mechanisms common to many neurodegenerative disorders, including oxidative stress, neuroinflammation, and neuronal death (Caputo et al. 2021; Piccialli, Tedeschi, et al. 2022; see Tables 1 and 2). Moreover, due to its ability to interfere with neurotransmissions, linalool and its derivatives have displayed anticonvulsant, anxiolytic, and anti‐depressant activities comparable to those of existing commercial medications, often with lower adverse effects (Thornton et al. 2020; Lee et al. 2018; Souto‐Maior et al. 2011; Linck et al. 2010; Cheng et al. 2014).

**TABLE 1:** Neuropharmacological effects of linalool tested in in vitro and ex vivo models.

Main finding	System/assay	Potency/tested concentration	References
Non‐competitive inhibition of [^3^H]MK801 binding; decreased *V*_max_ without changes in *K*_*d*_; no effect on [^3^H]muscimol binding	Mouse cortical membranes (ex vivo), radioligand binding assays ([^3^H]MK801, [^3^H]muscimol)	0.1–5 mM; IC_50_ = 2.97 ± 0.13 mM	Brum, Elisabetsky, and Souza (2001)
Competitive, dose‐dependent inhibition of [^3^H]glutamate binding	Rat cortex membranes (ex vivo), [^3^H]glutamate binding assay	0.1–5 mM; IC_50_ = 0.57 ± 0.05 mM	Elisabetsky et al. (1999)
Inhibition of K^+^‐stimulated glutamate release and uptake; concentration‐dependent inhibition of MK‐801 binding	Cortical synaptosomes for glutamate release/uptake; mouse cortical membranes (ex vivo), radioligand binding assays ([^3^H]MK801, [^3^H]muscimol)	0.1–3.0 mM; IC_50_ (uptake) = 1.50 ± 0.08 mM	Brum, Emanuelli, et al. (2001)
Neuroprotection against glutamate‐induced excitotoxicity via reduced oxidative stress and mitochondrial dysfunction	Hippocampal slices exposed to NMDA (ex vivo); HT‐22 cells exposed to glutamate	100 μM	Sabogal‐Guáqueta et al. (2019)
Positive allosteric modulation of GABA_A_ receptors: potentiation of GABA‐evoked currents	Xenopus oocytes expressing GABA_A_ receptors (GABA application)	*Kᵢ* = 59 μM; *V*_max_ = 296%	Kessler et al. (2014); Hossain et al. (2002)
GABA‐mimetic inward currents, partially suppressed by picrotoxin; glycine‐mimetic responses also observed	Substantia gelatinosa neurons (juvenile mice, patch‐clamp; application of GABA or glycine)	—	Phuong et al. (2021)
Dose‐dependent reduction in respiratory burst frequency and inhibition of pre‐inspiratory neuron activity; increased inhibitory postsynaptic potentials; abolished by bicuculline	Brainstem–spinal cord preparations from neonatal rats (P0–P3) (ex vivo)	0.2–1 mM (dose‐dependent)	Shibuya et al. (2024)
Concentration‐dependent, reversible block of voltage‐gated Na^+^ current and peripheral nerve excitability; reduced CAP amplitude; increased rheobase and chronaxy	Dissociated dorsal root ganglion neurons and sciatic nerve preparation (ex vivo) (stimulated with KCl/current injection)	IC_50_ (CAP amplitude) = 0.78 ± 0.04 mM; tested 0.1–6 mM; at 0.8 mM ↑ rheobase and chronaxy	Leal‐Cardoso et al. (2010)
Suppressed protein aggregation; inhibited Aβ42 fibril formation; direct binding to amyloid fibrils via hydrophobic interactions with Lys45, Lys46, Glu57 confirmed by docking simulations	Aβ_42_ peptide aggregation assays (DTT‐ and thermal‐induced; Thioflavin T binding)	5 mM	Singh et al. (2024)

Abbreviations: μM, micromolar; Aβ42, amyloid beta 1‐42; CAP, compound action potential; DTT, dithiothreitol; GABA, gamma‐aminobutyric acid; GABAA, gamma‐aminobutyric acid type A receptor; Glu, glutamate; IC50, half maximal inhibitory concentration; *K*_*d*_, equilibrium dissociation constant; *K*_*i*_, inhibition constant; Lys, lysine; mM, millimolar; NMDA, *N*‐methyl‐d‐aspartate; P0–P3, postnatal days 0–3; *V*_max_, maximum velocity.

**TABLE 2:** Neuropharmacological effects of linalool tested in in vivo models.

Main finding	Model	Route of administration	Potency/tested dose	Assay	References
Anticonvulsant in Dravet zebrafish; inactive in PTZ‐induced seizures	Zebrafish larvae (WT and scn1Lab^−/−^, 5–6 dpf)	Waterborne exposure (24 h)	0.25–4 μM	Locomotion/seizure assays (PTZ; genetic DS model)	Thornton et al. (2020)
Delayed onset of NMDA‐induced clonic convulsions, without preventing seizures	CF‐1 mice, NMDA‐induced seizure model (270 mg/kg s.c.)	Intraperitoneal	350 mg/kg (ED_50_ vs. PTZ = 243 mg/kg, 186–304)	Behavioral seizure test	Elisabetsky et al. (1999)
Dose‐dependent inhibition of quinolinic acid‐induced seizures	CF‐1 mice, quinolinic acid‐induced seizures (9.2 mM i.c.v.)	Intracerebroventricolare	15–45 mM (ED_50_ = 16.9 mM, 0.8–24.5)	Behavioral seizure test	Elisabetsky et al. (1999)
Partial inhibition and delay of clonic convulsions; no effect on PTZ‐kindling‐induced increase in cortical glutamate binding	CF‐1 mice, PTZ‐kindling model	Oral	2.2–2.5 g/kg, every 3 days ×6	Behavioral seizure test	Elisabetsky et al. (1999)
Sedative effects; potentiated diazepam activity	Chicks	Oral	50 mg/kg	Sedation test; diazepam co‐administration	Islam, Al Hasan, Ferdous, Mia, et al. (2025)
Reduced sleep latency; prolonged sleep duration	Mice, thiopental sodium‐induced sleep model	Intraperitoneal	50 mg/kg	Sleep latency/duration after thiopental sodium	Islam, Al Hasan, Ferdous, Yana, et al. (2025)
Anxiolytic‐like effects; abolished in anosmic mice; flumazenil‐sensitive	Male C57BL/6N mice	Inhalation (odor exposure)	20, 200, 2000 μL vapor	Light/dark box; elevated plus maze; rotarod	Harada et al. (2018)
Anxiolytic in females after repeated exposures; borderline in males; synergism with CBD	Male and female C57BL/6J mice	Vapor inhalation (short pulls)	3 s or 6 s pulls; ± CBD 30 mg/mL	Elevated plus maze	Wagner et al. (2024)
Normalized stress‐induced gene expression; ↑ Oxt, Npy; restored neurotransmitter markers	Male Wistar rats, restraint stress (2 h)	Inhalation (20 μL in 40 L chamber)	~0.5 ppm vapor	Gene expression analysis (hypothalamus)	Yoshida et al. (2017)
Stress‐relieving; ↑ locomotion and central entries; ↑ DA, ACh, GABA	Female C57 mice	Inhalation (nanoparticle‐coated silk)	Continuous exposure, 7 days	Open field test; neurotransmitter analysis	Lu et al. (2020)
Reversed METH‐induced neurotoxicity; normalized behavior, antioxidants, neurotransmitters, inflammation, proteome	Rats, methamphetamine neurotoxicity model	Oral	25 or 50 mg/kg	Behavioral assays (sucrose preference, open field, forced swim, tail suspension); biochemical assays	Koriem and El‐Qady (2023)
Neuroprotection against Cd‐induced hippocampal damage; restored antioxidants; ↓NF‐κB and Caspase‐3	Male Sprague–Dawley rats, CdCl_2_ neurotoxicity model	Intraperitoneal	100 mg/kg/day ×14 days (*C*_*d*_ 3 mg/kg/day ×7 days)	Oxidative stress and histopathology	Kaya et al. (2025)
Prevented RF‐EMR–induced deficits; preserved excitability, LTP, antioxidant balance; normalized trace elements	Pregnant Wistar rats (offspring assessed), prenatal RF exposure model	Oral gavage	25 mg/kg/day ×21 days	Electrophysiology (CA1 neurons); oxidative stress; behavioral tests	Azimzadeh and Noorbakhshnia (2024)
Suppressed epileptiform activity at low conc; induced excitability at high conc	Snail neurons (*Caucasotachea atrolabiata*)	Bath application	0.1–0.4 mM	Electrophysiology (spontaneous and PTZ‐induced activity)	Vatanparast et al. (2017)
Cannabimimetic behaviors; weak antinociception; hypothermia, catalepsy, hypolocomotion	CD‐1 mice	Intraperitoneal	50–200 mg/kg	Cannabinoid tetrad; tail‐flick test	LaVigne et al. (2021)
Cannabimimetic effects; antinociception in neuropathic/inflammatory pain; synergism with morphine; A2A receptor involvement	CD‐1 mice, pain models (paclitaxel, LPS)	Intraperitoneal	100–200 mg/kg; ± morphine 10 mg/kg	Acute and chronic pain assays; conditioned place preference	Schwarz et al. (2024)
Improved memory; reduced apoptosis and oxidative stress; upregulated Nrf2/HO‐1	C57BL/6J mice, Aβ1–40 hippocampal injection model	Intraperitoneal	50 and 100 mg/kg	Morris water maze; passive avoidance; histology; biochemical assays	Xu et al. (2017)
Improved learning and spatial memory; enhanced risk assessment; reduced β‐amyloidosis, tau hyperphosphorylation, gliosis, and pro‐inflammatory markers (p38 MAPK, NOS2, COX‐2, IL‐1β)	3xTg‐AD mice, 18–21 months	Oral	25 mg/kg every 48 h × 3 months	Morris water maze; elevated plus maze; histology; biochemical assays	Sabogal‐Guáqueta et al. (2016)
Increased survival in Aβ42‐expressing flies; reduced apoptosis and neurodegeneration in brain and eye discs; decreased ROS, NO, and inflammation; no change in Aβ levels or aggregation	*Drosophila melanogaster* (elav‐GAL4 > UAS‐Aβ42; GMR‐GAL4 > UAS‐Aβ42)	Dietary feeding	200–800 μM	Survival; histology; oxidative stress markers	Yuan et al. (2021)
Reduced oxidative stress (↓4‐HNE) and gliosis (↓GFAP) in hippocampus; attenuated neurodegeneration induced by Aβ	Sprague–Dawley rats with hippocampal Aβ1–42 injection	Intraperitoneal	50 and 100 mg/kg	Histology; oxidative stress markers	Yuan et al. (2021)
Reduced hippocampal amyloid deposits induced by intracerebroventricular Aβ42 oligomers, comparable to 4‐PBA	Male Sprague–Dawley rats with i.c.v. Aβ42 oligomers	Intraperitoneal	—	Thioflavin T histology of hippocampus	Singh et al. (2024)
Decreased immobility time in forced swim test; effect blocked by 5‐HT1A antagonist and modified by α2‐adrenergic antagonist, not by other monoaminergic blockers	Adult male ICR mice (forced swim test)	Intraperitoneal	100 mg/kg × 3 months	Forced swim test with pharmacological antagonists	Guzmán‐Gutiérrez et al. (2015)
Increased withdrawal latency in hot plate test; reduced formalin‐induced acute and inflammatory pain behaviors; effects abolished by anosmia or orexin deficiency; associated with orexin neuron activation	C57BL/6 mice (anosmic and orexin‐deficient mutants)	Inhalation (vapor)	1%, 10%, 100% vapor, 5–60 min	Hot plate; formalin test; c‐Fos immunohistochemistry	Tashiro et al. (2016)

Abbreviations: 4‐HNE, 4‐hydroxynonenal; μM, micromolar; 5‐HT1A, serotonin receptor 1A; ACh, acetylcholine; Aβ, beta‐amyloid; CBD, cannabidiol; Cd, cadmium; CdCl_2_, cadmium chloride; COX‐2, cyclooxygenase‐2; DA, dopamine; dpf, days post fertilization; ED_50_, median effective dose; GABA, gamma‐aminobutyric acid; GFAP, glial fibrillary acidic protein; HO‐1, heme oxygenase‐1; i.c.v., intracerebroventricular; i.p., intraperitoneal; IL‐1β, interleukin‐1 beta; LTP, long‐term potentiation; MAPK, mitogen‐activated protein kinase; METH, methamphetamine; mg/kg, milligrams per kilogram; mM, millimolar; NF‐κB, nuclear factor kappa‐light‐chain‐enhancer of activated B cells; NO, nitric oxide; NOS2, nitric oxide synthase 2; Npy, neuropeptide Y; Oxt, oxytocin; ppm, parts per million; RF‐EMR, radiofrequency electromagnetic radiation; ROS, reactive oxygen species; s.c., subcutaneous; WT, wild type.

Apart from being absorbed from skin, linalool was shown to be absorbed from the gut following oral administration and excreted almost completely (95%) within 48 h (Cal 2006; Parke et al. 1974). In healthy humans, inhalation of linalool resulted in higher plasma levels in comparison with dermal application, with a maximum level of 72.7 ng/mL observed after 40–45 min post‐exposure (Friedl et al. 2010). Even if definite analyses of linalool brain levels following administration are still few, linalool is able to cross the blood brain barrier (BBB) due to a low molecular weight and high lipophilicity. Accordingly, a study investigating the transfer of fragrant molecules to the brain by gas chromatography–mass spectrometry found that linalool was maximally transferred to the brain after 90 min of inhalation (Satou et al. 2010).

### 3.1 Effects of Linalool on Glutamatergic Transmission

The ability of linalool to interfere with glutamatergic transmission has been reported. It was shown to inhibit [^3^H]MK801 binding at the NMDA receptor complex and [^3^H]glutamate binding in cortical membranes (Brum, Elisabetsky, and Souza 2001; Elisabetsky et al. 1999). This mechanism seemed to underlie the anticonvulsant properties exerted by linalool in different seizure models. Linalool was indeed able to delay the onset of NMDA‐induced seizures and to protect against convulsions induced by quinolinic acid administration, as well as to partially inhibit and significantly delay pentylenetetrazol (PTZ)‐kindling (Elisabetsky et al. 1999). Interestingly, linalool was also shown to inhibit the K^+^‐stimulated glutamate release and glutamate uptake by mouse cortical synaptosomes. In addition, it displayed the ability to inhibit the binding of MK801, a NMDA antagonist with anticonvulsant properties, in a concentration‐dependent manner (Brum, Emanuelli, et al. 2001). Together these findings suggest the ability of linalool to block NMDA receptors in vitro and in vivo, even if further investigations are needed to confirm this mechanism. Of note, due to its antioxidant properties, linalool also proved to be neuroprotective against excitotoxicity since it reduced oxidative stress and mitochondrial dysfunction in hippocampal slices exposed to NMDA and in HT‐22 cells exposed to glutamate, hence displaying the potential to serve as a multi‐target therapeutic agent (Sabogal‐Guáqueta et al. 2019).

### 3.2 Effects of Linalool on GABAergic Transmission

Linalool and linalool‐rich EOs have displayed sedative and anxiolytic‐like effects (López et al. 2017; Linck et al. 2010, 2009; Gastón et al. 2016; Rombolà et al. 2017). Therefore, the capability of the monoterpene to affect the GABAergic transmission has been investigated. Linalool was found to significantly potentiate GABA‐evoked currents acting on GABA_A_ receptors (Kessler et al. 2014; Hossain et al. 2002). Although such an effect might be mediated by linalool binding to the potentiation site of the receptor and by the subsequent increase of GABA‐binding affinity, further investigations are needed to confirm this mechanism. Interestingly, Phuong et al. showed that linalool was able to exert a GABA‐mimetic effect by inducing inward currents that were partially suppressed by picrotoxin, a well‐known GABA_A_ receptor antagonist (Phuong et al. 2021). Additional mechanistic evidence comes from an ex vivo study by Shibuya et al., in which direct application of linalool to brainstem–spinal cord preparations from neonatal rats (P0–P3) led to a dose‐dependent reduction in respiratory burst frequency and inhibition of pre‐inspiratory neuron activity. These effects were abolished by the GABA_A_ receptor antagonist bicuculline and accompanied by an increase in inhibitory postsynaptic potentials, supporting a direct modulatory action of linalool on central GABAergic transmission (Shibuya et al. 2024).

Linalool appears to exert its GABA‐mediated effects through multiple neuroanatomic pathways, depending also on the route of administration. Notably, sedative and anxiolytic‐like effects have been observed following both oral and intraperitoneal administration of linalool (Linck et al. 2010; Islam, Bhuia, Mostakim, Chowdhury, et al. 2025). In a study by Islam and colleagues, the oral administration of linalool (50 mg/kg) in chicks induced clear sedative effects and enhanced the pharmacological activity of diazepam when co‐administered, suggesting possible additive or synergistic interaction within the GABAergic system (Islam, Al Hasan, Ferdous, Mia, et al. 2025). Similarly, the intraperitoneal administration of linalool significantly reduced sleep latency and prolonged sleep duration in thiopental sodium‐injected mice (Islam, Al Hasan, Ferdous, Yana, et al. 2025). *In silico* analyses from the same research group revealed a moderate binding affinity of linalool toward GABA_A_ receptor subunits α3 (−5.0 kcal/mol), α1 and β2 subunits (−6.8 kcal/mol) (Islam, Al Hasan, Ferdous, Mia, et al. 2025), supporting the hypothesis that GABA_A_ receptors play a central role in the observed effects.

The effects of linalool inhalation have been extensively investigated, particularly regarding the routes by which it reaches or influences the central nervous system. Inhaled linalool may either be transmitted to the CNS by olfactory neural pathways, hence bypassing the BBB, or undergo respiratory absorption to reach the CNS via the bloodstream (Hanson and Frey 2008). Alternatively, it can act as an olfactory stimulator, triggering signaling cascades in subcortical structures through the olfactory system (Soudry et al. 2011). Some authors reported that the anxiolytic‐like effects of linalool were blocked by the benzodiazepine antagonist flumazenil and occurred only in mice with an intact olfactory system, suggesting that a combined involvement of GABA_A_ receptor modulation and olfactory pathways is necessary (Harada et al. 2018). Nevertheless, opposite results have been reported for lavender EO, as its anxiolytic‐like effect, which is mainly mediated by linalool, was instead preserved in anosmic mice (Chioca et al. 2013). In this regard, recent findings by Wagner et al. (2024) suggested that the contribution of olfactory pathways to the anxiolytic effects of linalool may be both sex‐ and exposure‐dependent. Inhaled linalool produced clear anxiolytic‐like effects in female mice after repeated exposures, but had opposite, anxiogenic effects in males unless administered briefly, suggesting a non‐linear relationship (Wagner et al. 2024). These differences were paralleled by sex‐specific olfactory responses and support the idea that the olfactory system can mediate divergent outcomes depending on sensitivity, as well as exposure duration and frequency.

Complementary data in humans come from Pei et al. (2024), who assessed the effects of linalool inhalation following cognitive stress. While no significant changes were observed in subjective anxiety scores, participants exposed to linalool showed a clear autonomic and neurophysiological shift toward relaxation. Specifically, linalool reduced systolic blood pressure and heart rate post‐task and significantly increased delta, theta, and alpha EEG activity in frontal, parietal, and occipital areas, a pattern typically associated with decreased arousal and emotional recovery (Pei et al. 2024). Although the lack of subjective effects might reflect the specific experimental conditions or dose used, the convergence of physiological and cortical markers strongly supports the hypothesis of a central, likely GABAergic, mechanism. Importantly, these findings reinforce the idea that linalool can modulate stress responses via olfactory and systemic routes, even in the absence of conscious awareness.

Supporting a central action of linalool, Yoshida et al. (2017) found that linalool inhalation returned GABAergic transmission to a normal state by restoring gene expression in the hypothalamus after a stress‐induced perturbation. In particular, linalool inhalation was able to up‐regulate the glutamate decarboxylase 1‐encoding gene (*Gad1*), among others (Yoshida et al. 2017). In line with this, Lu et al. (2020) demonstrated that linalool‐loaded nanoparticles led to significantly increased GABA levels in the brain of the treated mice. These findings are also consistent with those reported by Koriem and El‐Qady (2023) showing that oral linalool administration counteracted methamphetamine‐induced neurotoxicity in the hypothalamus by normalizing neurotransmitter levels, restoring antioxidant enzyme activity, and reducing neuroinflammation and neuronal degeneration. Although not specifically focused on GABAergic modulation, this study supports a central action of linalool in preserving hypothalamic homeostasis under methamphetamine‐induced neurotoxic stress. Similarly, Kaya et al. (2025) demonstrated that linalool protects against cadmium‐induced hippocampal neurodegeneration by attenuating oxidative stress, downregulating NF‐κB activation, and suppressing apoptosis. The protective effect, linked to the 4‐HNE/NF‐κB signaling axis, reinforces the notion of a multi‐target central action with anti‐inflammatory and cytoprotective properties (Kaya et al. 2025). Further supporting a central neuroprotective role, Azimzadeh and Noorbakhshnia (2024) reported that maternal linalool administration in rats prevented anxiety‐like behavior, memory deficits, and synaptic plasticity impairments in adolescent offspring exposed to prenatal radiofrequency radiation. The treatment preserved hippocampal long‐term potentiation and counteracted alterations in trace element homeostasis (Azimzadeh and Noorbakhshnia 2024). Although not focused on GABAergic signaling, these findings are consistent with a broader modulatory effect of linalool on E/I balance and stress‐induced neural dysfunction.

In nature, but also after intake by ingestion or inhalation, linalool undergoes numerous chemical modifications including oxygenation and acetylation, which may occur through EO autoxidation or exposition to the enzymatic activity of cytochrome P‐450 (Meesters et al. 2007). Such modifications may significantly affect the pharmacological properties of linalool, including the modulatory activity on GABA_A_ receptor function. For example, Milanos et al. (2017) showed that acetylated and oxygenated linalool derivatives including linalyl acetate no longer potentiated GABAergic currents. Similarly, oxygenated linalool derivatives lost the ability to potentiate GABAergic responses in a significant manner, suggesting a loss of GABA_A_‐related activity upon metabolic conversion (Milanos et al. 2017).

### 3.3 Effects of Linalool on Ion Channels and Intrinsic Neuronal Excitability

In addition to the well‐documented alterations in synaptic transmission, increasing evidence highlights changes in intrinsic neuronal excitability as a major contributor to the hyperexcitable phenotype observed in AD. This form of excitability, primarily governed by the function and distribution of voltage‐gated ion channels, plays a crucial role in regulating neuronal firing properties. As discussed above, Aβ pathology has been implicated in the dysregulation of multiple ion channel subtypes, including the aberrant upregulation of Na_V_1.6 currents and the downregulation of Na_V_1.1, among others. The convergence of intrinsic excitability dysfunction with disrupted excitatory and inhibitory synaptic inputs promotes widespread hyperexcitability and enhanced susceptibility to network hypersynchrony, a feature increasingly recognized as central to both cognitive decline and subclinical seizure activity in AD.

Within this pathological context, the ability of linalool to modulate various classes of ion channels acquires particular relevance. In peripheral sensory neurons, linalool has been shown to block Na_V_ currents in a dose‐dependent manner via a direct, non‐competitive mechanism of action, resulting in reduced neuronal firing—a profile reminiscent of local anesthetics, though with lower potency (Leal‐Cardoso et al. 2010). Further support for an inhibitory effect on neuronal excitability comes from invertebrate models, where linalool reversibly reduced the spontaneous firing rate of central neurons, potentially through interactions with Na^+^ and/or K^+^ channels (Vatanparast et al. 2017). In two zebrafish models of epileptiform activity, one chemically induced and the other genetically driven via a mutation in the *scn1a* gene (encoding Na_V_1.1), linalool significantly attenuated seizure‐like behaviors, confirming its anticonvulsant properties (Thornton et al. 2020). Although these models are not specific to AD, the observed efficacy in a Na_V_1.1‐deficient background is noteworthy, given the documented downregulation of this channel in AD‐related interneuron dysfunction. While direct evidence of linalool's action on neuronal K^+^ currents is still lacking, studies on vascular tissues have shown that linalool‐induced vasorelaxation is partially blocked by tetraethylammonium, hinting at a possible activation of K^+^ channels (Kang and Seol 2015). In line with this, lavender EO has been shown to activate KCNQ5 channels expressed in smooth muscle cells, contributing to vascular tone modulation (Redford and Abbott 2022).

Oxidative stress is recognized as a central modulator of ion channel function in AD, contributing to the pathophysiology of channelopathies that underlie intrinsic neuronal hyperexcitability. Among others, K_V_2.1 and several K^+^ channels are known to be redox‐sensitive as they can undergo functional alterations in response to elevated reactive oxygen species (ROS) production, a process tightly linked to Aβ pathology (Frazzini et al. 2016; Pannaccione et al. 2005; Piccialli, Tedeschi et al. 2021). Under conditions of oxidative stress, these channels may become dysfunctional, contributing to impaired neuronal repolarization and increased excitability (Frazzini et al. 2016; Pannaccione et al. 2005; Cotella et al. 2012). Linalool has been shown to exert notable antioxidant effects, reducing intracellular ROS levels in both neuronal and peripheral models, enhancing endogenous antioxidant systems such as superoxide dismutase and catalase, and attenuating markers of lipid peroxidation and inflammation (e.g., TNF‐α, IL‐1β) (Alserhani et al. 2025). It is therefore possible to speculate that the antioxidant activity of linalool may contribute to the stabilization of redox‐sensitive ion channels. This represents a potentially relevant indirect mechanism by which linalool could mitigate neuronal hyperexcitability and support the restoration of E/I balance.

Altogether, these findings highlight linalool as a multifaceted compound with the ability to modulate both neurotransmissions and intrinsic neuronal excitability through direct or indirect actions on key voltage‐gated channels. Such properties are highly relevant to restoring E/I balance in the early stages of AD, where neuronal hyperexcitability and interneuron dysfunction represent critical and potentially druggable contributors to cognitive decline. However, further targeted pharmacological investigations are warranted to elucidate whether linalool can modulate intrinsic excitability in specific neuronal populations, particularly GABAergic interneurons and hippocampal pyramidal cells, expressing ion channel isoforms (e.g., Na_V_1.1, K_V_2.1) known to be critically altered in AD‐related E/I imbalance.

### 3.4 Additional Targets of Linalool: CB1 and A2A Receptors

Given the growing interest in terpenes as active constituents of cannabis extracts, several studies have investigated the interaction of linalool, along with other cannabis‐derived terpenes, with cannabinoid and adenosine receptors, the latter being increasingly implicated in the broader pharmacological effects of cannabis phytocomplexes.

A study by LaVigne et al. (2021) recently described linalool and other terpenes as “cannabimimetic,” as they induced at least three of the four classical cannabinoid tetrad behaviors: hypolocomotion, hypothermia, catalepsy, and analgesia (Metna‐Laurent et al. 2017). The antinociceptive effect was shown to be CB1‐dependent, as pretreatment with rimonabant, a selective CB1 inverse agonist, abolished the terpene‐induced response. Moreover, combining terpenes with WIN55,212‐2 (a CB1 agonist) enhanced the effect beyond either treatment alone, suggesting terpene–cannabinoid synergy. Hypothermic responses were also additive, although largely CB1‐independent, suggesting that terpenes, like cannabinoids, may act through both CB1‐dependent and CB1‐independent mechanisms. Terpene‐induced catalepsy and hypolocomotion were partially mediated by adenosine A2A receptors. Interestingly, linalool showed sex‐specific pharmacodynamics: although it produced comparable antinociceptive effects in both sexes when administered alone, it had a stronger additive effect in males, whereas females showed a delayed and non‐additive response. In the case of hypolocomotion, CB1 mediated the effect in males, while A2A receptors appeared more involved in females. The same study confirmed in vitro that linalool and other terpenes interact with CB1 receptors, albeit with low potency (LaVigne et al. 2021). Nonetheless, direct CB1 agonism by linalool remains unconfirmed. Alternative mechanisms, such as interactions with membrane microdomains facilitating CB1 activation or modulation of endocannabinoid synthesis/degradation, should be considered.

Additional support for A2A‐mediated mechanisms comes from studies in murine models of chronic pain and neuroinflammation, where linalool and other cannabis‐derived terpenes such as β‐caryophyllene, α‐humulene, and geraniol induced robust antinociceptive effects. These responses were fully abolished by pretreatment with istradefylline, a selective A2A receptor antagonist, and were not accompanied by significant locomotor impairment or reward‐related behaviors, suggesting a favorable therapeutic profile (Schwarz et al. 2024). Notably, these effects occurred independently of CB1 activation, and in one study, no synergy with phytocannabinoids was observed, whereas additive effects were reported in combination with morphine. These findings point to a distinct, CB1‐independent mechanism by which linalool may modulate pain perception and inflammatory signaling through adenosinergic pathways (Schwarz et al. 2024).

### 3.5 Neuroprotective Effects of Linalool in AD Models

Although evidence has shown that linalool can rescue memory impairment in preclinical models of AD, these effects have been mainly interpreted in relation to its anti‐inflammatory and antioxidant properties, and to its ability to reduce pathogenic proteins such as Aβ and tau, while its potential impact on synaptic function and E/I balance has not been thoroughly investigated. A study by Xu et al. (2017) reported that linalool significantly attenuated cognitive deficits induced by Aβ in Aβ_1‐40_‐injected mice. In particular, the intraperitoneal administration of linalool (100 mg/kg) was able to improve cognitive performance in Morris water maze and step‐through tests, while it reversed the Aβ_1‐40_‐induced cell injury at the hippocampal level, a result that was correlated to the reduction of oxidative stress and apoptosis (Xu et al. 2017). Similarly, the oral administration of linalool (25 mg/kg) for 3 months improved learning and spatial memory in 3xTg‐AD 21‐ to 24‐month‐old mice. In addition, linalool‐treated 3xTg‐AD mice displayed a significant reduction in β‐amyloidosis, tauopathy, and gliosis in the hippocampus and amygdala, as well as significantly decreased levels of the pro‐inflammatory markers p38 MAPK, NOS2, COX2, and IL‐1β (Sabogal‐Guáqueta et al. 2016). The intraperitoneal administration of linalool was also found to ameliorate the AD‐like phenotype in Aβ_1‐42_‐injected rats due to its antioxidant and anti‐inflammatory activities (Yuan et al. 2021). Recent data further suggest that linalool may exert direct anti‐amyloidogenic effects. Singh et al. (2024) demonstrated that linalool acts as a chemical chaperone by inhibiting the aggregation of Aβ_1‐42_ both in vitro and in vivo. In thioflavin T fluorescence assays, linalool significantly reduced fibril formation, while docking studies revealed stable hydrophobic interactions with key Aβ residues involved in self‐assembly. In a rat model of Aβ_1‐42_‐induced neurotoxicity, linalool administration led to a marked reduction in hippocampal amyloid deposition, indicating its potential to interfere with the early stages of amyloid fibrillogenesis (Singh et al. 2024). In line with this, Stylianopoulou et al. (2023) reported a similar anti‐aggregation activity of linalool in a cell‐based biosensor system for α‐synuclein, suggesting that linalool may target shared structural features of aggregation‐prone proteins across neurodegenerative diseases (Stylianopoulou et al. 2023).

## 4 Lavender

*Lavandula angustifolia* Mill. (hereafter referred to as lavender) is a plant of the Lamiaceae family, which comprises different species including, among others, *Lavandula latifolia* Mill., *Lavandula Stoechas* Mill., and *Melissa officinalis* L. Lavender contains a huge number of medicinal compounds, such as terpenes, alcohols, polyphenols, coumarins, flavonoids, and others. Linalool and its ester form, linalyl acetate, are the main volatile constituents of lavender EO (Buchbauer et al. 1991). Nonetheless, the chemical composition of lavender EO may vary in its psychoactive constituents due to environmental factors, genetic differences, and extraction methods. To address this variability, the International Standard Organization defined specific standards for the extraction and analytical composition of lavender oil to establish EO quality grades (ISO 3515:2002). The European Medicine Agency Committee on Herbal Medicine Products assessed that lavender oil may exert positive effects on several clinical conditions. Both lavender and its EO have been investigated through various administration routes, including inhalation, oral intake, and aromatherapy (Sayed et al. 2020), and have consistently shown multiple beneficial properties, particularly sedative and anxiolytic‐like effects, as demonstrated in both preclinical and clinical studies (Linck et al. 2010; Greenberg and Slyer 2018; Dold et al. 2023; Kasper and Eckert 2025; Uehleke et al. 2012; see Table 3). The inhalatory administration is considered the most promising way to deliver the beneficial effects of lavender to the CNS, since the olfactory pathway is a direct and rapid route to the brain. Moreover, the nasal administration is less invasive than the oral or intravenous administration and avoids hepatic first metabolism, hence ensuring higher bioavailability.

**TABLE 3:** Studies investigating the effects of lavender preparations in humans.

Main finding	Population/condition	Route of administration	Dosage	References
Improved restlessness, anxiety, sleep disturbance, and depressed mood; reductions in depression and global symptom severity; improved mental health quality of life and sleep outcomes; mild gastrointestinal adverse events	Open‐label, non‐controlled, prospective clinical trial; 50 outpatients with neurasthenia, PTSD, or somatization disorder	Oral (Silexan)	80 mg/day for 6 weeks	Uehleke et al. (2012)
Increased N3 sleep proportion, reduced N2; enhanced delta power during N3; reduced alpha/beta activity during wake; improved subjective sleep quality and next‐day alertness	Single‐blind, randomized, crossover sleep‐laboratory study; 9 healthy young adults	Inhalation during sleep (odorless pulse system)	Nightly aroma release during sleep period	Ko et al. (2021)
Lavender odor during NREM enhanced slow‐wave and spindle activity; effects proportional to odor exposure; no effect during REM and no arousal responses	Randomized, single‐night, polysomnography study with odor exposure (parallel groups, not placebo‐controlled); 34 healthy adults	Inhalation during sleep (olfactometer, 5–20 s pulses)	Undiluted lavender oil, pulses every 9–15 min after stage‐2 onset	Perl et al. (2016)
Lavender aroma promoted greater left frontal EEG activation (improved mood regulation) in adults with right‐frontal baseline asymmetry; similar but weaker effects in infants	Experimental, randomized EEG studies, not blinded, placebo control absent; Study 1: 39 adults; Study 2: 27 infants	Inhalation (10% lavender oil in grapeseed oil)	Adults: 3 drops on cotton swab, 3 min; Infants: 3 drops above crib	Sanders et al. (2002)
Lavender extract induced reduced EEG power across frequency bands and increased subjective tiredness at 180 min, comparable to valerian but less than diazepam	Randomized, double‐blind, placebo‐controlled, crossover trial; 24 healthy adult women	Oral (lavender extract)	1200 mg single dose	Schulz et al. (1998)
Lavender oil massage reduced cortisol and daytime naps; blends with valerian/chamomile further improved insomnia, neuropsychiatric symptoms, and depression scores; no serious adverse effects	Open‐label, prospective pilot study; 13 elderly dementia patients with insomnia	Transdermal massage (3% lavender oil in jojoba)	Daily face and hand massage for 2 weeks	Lee et al. (2023)
Lavender olfactory training significantly reduced seizure frequency and duration; greater benefit in pediatric and focal epilepsy; quality of life and olfactory scores improved; no adverse effects	Prospective, open‐label, uncontrolled 3‐month preliminary trial; 24 patients with drug‐resistant epilepsy	Inhalation (lavender essence bottle, 2 cm from nose)	Twice daily, 30–45 s per session, 3 months	Yilmaz et al. (2022)
Silexan was non‐inferior to lorazepam for reducing anxiety; higher remission and response rates with Silexan; improved sleep; mild gastrointestinal adverse events only	Multicenter, randomized, double‐blind, lorazepam‐controlled trial; 77 adults with generalized anxiety disorder	Oral (Silexan)	80 mg/day for 6 weeks	Woelk and Schläfke (2010)
Meta‐analysis of five randomized controlled trials: Silexan significantly reduced somatic anxiety symptoms, insomnia, and fatigue; improved physical health‐related quality of life; safe and well tolerated	Systematic review and meta‐analysis of randomized, placebo‐controlled clinical trials; adults with generalized anxiety disorder or subthreshold anxiety disorders	Oral (Silexan)	80 mg/day for 10 weeks	von Känel et al. (2021)
Lavender aroma therapy significantly reduced neuropsychiatric symptoms in dementia, including hallucinations, agitation, irritability, and motor activity; no adverse events	Randomized, controlled (lavender vs. no treatment), open‐label trial; 28 patients with dementia	Inhalation (2 drops on collar, 3×/day)	4 weeks	Fujii et al. (2008)
Lavender aroma therapy for 4 weeks significantly reduced neuropsychiatric symptoms in frontotemporal lobar degeneration, especially apathy; symptoms worsened after discontinuation; reduced psychotropic use; no adverse events	Open‐label, uncontrolled pilot study; 20 patients with frontotemporal lobar degeneration	Inhalation (method not fully specified)	Daily for 4 weeks	Kimura and Takamatsu (2013)
No overall benefit versus placebo, but in dementia subgroup lavender reduced agitation and irritability; safe and well tolerated	Randomized, placebo‐ and active‐controlled, crossover trial; 49 aged care residents (39 with dementia, 10 without)	Inhalation (2 drops on patch, collar, 2 h/day)	14‐day crossover periods	Watson et al. (2019)
Home‐based lavender aromatherapy reduced disinhibition and irritability in dementia; improved quality of life; caregiver burden unchanged; mild adverse events only	Randomized, controlled, wait‐list control trial (caregiver‐delivered intervention); 80 older persons with dementia at home	Inhalation (2 drops on cotton pad, collar, 1 h twice daily)	3 weeks	Li et al. (2025)
Mixed aromatherapy (lavender evening plus rosemary/lemon morning) improved cognitive function in Alzheimer's patients; safe, no adverse effects	Open‐label, crossover design (control, intervention, washout); 28 elderly inpatients with dementia (17 ad, others mixed)	Inhalation (diffuser, rosemary/lemon AM, lavender/orange PM)	28 days	Jimbo et al. (2009)

Abbreviations: AD, Alzheimer's disease; CMAI, Cohen–Mansfield Agitation Inventory; EEG, electroencephalography; FTLD, Frontotemporal lobar degeneration; GDS‐SF, Geriatric Depression Scale—Short Form; HAM‐A, Hamilton Anxiety Rating Scale; HAM‐D, Hamilton Depression Rating Scale; ISI, Insomnia Severity Index; NPI, Neuropsychiatric Inventory; NPI‐Q, Neuropsychiatric Inventory Questionnaire; NREM, Non‐Rapid Eye Movement sleep; QoL, Quality of Life; RCT, Randomized Controlled Trial; REM, Rapid Eye Movement sleep; SCL‐90‐R GSI, Symptom Checklist‐90‐Revised Global Severity Index; SF‐36, 36‐Item Short Form Health Survey; TDAS, Touch Panel‐type Dementia Assessment Scale.

### 4.1 Effects of Lavender on Cognition, Sleep Regulation, and Their Relevance in AD

Although a systematic review of the impact of lavender EO inhalation on cognitive functions is still lacking, several authors have reported putative cognitive‐enhancing effects in healthy subjects, along with the well‐known anxiolytic properties. The improvement in cognitive performance by lavender has been linked to its influence on arousal and attention and to its sedative and calming properties (Uehleke et al. 2012; Shimizu et al. 2008). As linalool is the main component of lavender EO, its ability to interact with GABAergic circuits is considered a key mechanism underlying the neurobiological effects of lavender, including anxiolytic and cognitive‐related outcomes. Consistently, the sedative and anxiolytic effects of lavender EO have been directly correlated with plasma concentrations of linalool and linalyl acetate (3–11 ng/mL) (Buchbauer et al. 1993). Nonetheless, some studies comparing lavender EO to reference anxiolytic drugs suggest alternative or additional mechanisms involving NMDA receptors or serotonergic pathways (López et al. 2017; Chioca et al. 2013).

The cognitive effects of lavender EO may also involve olfactory‐mediated pathways. Several studies, for instance, have described anatomical and functional connectivity between the olfactory bulb and subcortical structures implicated in memory processes, suggesting that olfactory stimulation with specific EOs could modulate memory function (Zelano et al. 2016; Vasavada et al. 2017). Consistently, stimulation of olfactory epithelium mechanoreceptors has been shown to synchronize the electrical activity of different brain regions, including the olfactory bulb, hippocampus, and amygdala (Heck et al. 2019; Piarulli et al. 2018). Olfactory stimulation with certain EOs, particularly lavender, has been proposed to reduce agitation and promote autobiographical memory recall, especially when paired with emotionally salient cues. However, methodological heterogeneity and lack of olfactory screening remain significant limitations across studies (D'Andrea et al. 2022).

Intriguingly, a functional link has been proposed between the olfactory system and slow‐wave oscillations during sleep, potentially mediated by odor‐evoked responses and respiration rhythm (Fontanini and Bower 2006; Gaeta and Wilson 2022). For this reason, several studies have explored the use of olfactory stimulation as a strategy to improve sleep quality (Gaeta and Wilson 2022). A recent preclinical study using polysomnography in freely moving mice showed that lavender EO administered via inhalation during the inactive phase significantly increased non‐REM sleep, reduced sleep latency, and enhanced delta activity in the EEG. These effects were mediated by GABAergic neurons in the central amygdala and were abolished by olfactory interruption by zinc sulfate injection or central amygdala GABAergic neuron inhibition, underscoring the involvement of an olfactory–limbic circuit in sleep regulation (Ren et al. 2025). Nonetheless, a multifaceted mechanism involving cholinergic and histaminergic systems—not only GABAergic—has been proposed for the sleep‐improving properties of lavender EO (Xu et al. 2023).

Clinical findings support these experimental results. Lavender aroma has indeed been reported to promote low‐frequency (theta and delta) brain waves and to influence sleep architecture in humans (Ko et al. 2021; Perl et al. 2016). Notably, the effect appears to be direct and not mediated by expectancy or emotional valence, nor does it involve EEG arousal markers such as K‐complexes or trigeminal activation.

Given the known coupling between respiratory rhythm and cortical slow‐wave oscillations during sleep, this mechanism may contribute to the neurophysiological effects of lavender on sleep architecture and EEG patterns. In this regard, recent data from Shibuya et al. (2024) showed that linalool directly modulates the respiratory rhythm generator in the brainstem from neonatal rats, exerting a dose‐dependent inhibitory effect through the activation of GABA_A_ receptors. Specifically, linalool reduced the firing of pre‐inspiratory neurons and stabilized the respiratory pattern, an effect not observed with linalyl acetate (Shibuya et al. 2024). Although the study was not focused on sleep, these findings support the idea that linalool—and lavender EO—can influence central autonomic circuits implicated in the regulation of arousal states.

Overall, the available data demonstrate that lavender EO, particularly when administered via inhalation, improves subjective sleep quality in various populations, including older adults and menopausal women, with a favorable safety profile (Luo and Jiang 2022). On this basis, recent studies have begun to elucidate the neurobiological mechanisms through which lavender and linalool may regulate sleep architecture and arousal states. A recent EEG study found that lavender EO inhalation elicited brain wave responses partially similar to those induced by diazepam, while also producing distinct and specific effects on sleep–wake patterns (Manor et al. 2021). These findings suggest that the action of lavender is mediated by mechanisms that may potentially exert synergistic effects on sleep regulation, such as olfactory stimulation and direct pharmacological modulation of the GABAergic system.

Of note, recent findings suggested that the effects of linalool on sleep architecture might not rely exclusively on GABAergic modulation. Some studies have reported that linalool can also influence serotonergic transmission, potentially through increased 5‐HT availability or enhanced activation of 5‐HT₁A receptors, as suggested by the reversal of its anxiolytic‐like effects following the administration of serotonergic antagonists in animal models (Guzmán‐Gutiérrez et al. 2015). Supporting this, a study comparing the effects of aromatherapy with five herbs linalool‐rich EOs in mice with parachlorophenylalanine‐induced insomnia suggested the ability of linalool to regulate the expression of both GABA_A_Rα1 and 5‐HT receptors (Feng et al. 2024).

Emerging evidence also indicates that linalool may activate orexinergic neurons in the lateral hypothalamus, an effect seemingly mediated through olfactory input rather than direct receptor binding (Tashiro et al. 2016). Notably, this neuronal activation was not associated with elevated corticosterone levels, suggesting the absence of a stress‐related response and pointing instead to a neurovegetative modulation. Intriguingly, while hyperactivity of orexin signaling has been implicated in the sleep disturbances commonly observed in AD, a finely tuned modulation of this system, rather than its suppression, could potentially aid in stabilizing circadian rhythms and promoting daytime alertness. Altogether, these observations suggest that linalool may engage broader neuromodulatory circuits beyond GABA, warranting further investigation into the potential of lavender EO for modulating sleep–wake dynamics.

Given that the disruption of slow‐wave oscillations during sleep has been linked to the development and worsening of memory deficits in AD (Gills and Bubu 2024; Pulver et al. 2024; Kollarik et al. 2022), the ability of lavender to affect these processes warrants closer examination. Indeed, experimental studies in rodent models have shown that chronic sleep restriction is associated with genetic and structural changes in the brain, including reduced hippocampal neuron spine density and impaired neurogenesis, with a profound impact on hippocampal function and consequent learning and memory impairment (Owen and Veasey 2020). Moreover, a direct correlation between poor sleep and increased brain Aβ levels has been demonstrated, likely due to the dysfunctional clearance mechanisms induced by poor sleep quality and duration (Boespflug and Iliff 2018; Mander et al. 2016; Kang et al. 2009). Overall, growing evidence links sleep disturbances to glymphatic dysfunction and neuroinflammation, reinforcing the idea that sleep restoration may positively influence AD progression. In line with this, experimental strategies aimed at improving slow‐wave activity and sleep, such as those targeting GABAergic interneurons, have been shown to ameliorate neuropathological and behavioral symptoms in AD models (Katsuki et al. 2022; Zhao et al. 2023).

Of note, the ability of lavender to produce EEG changes during sleep and improve sleep quality supports its use as a non‐pharmacological alternative to hypnotics like benzodiazepines, which are associated with several neurological risks (Ko et al. 2021; Manor et al. 2021; Sanders et al. 2002; Schulz et al. 1998). This issue is particularly relevant considering that sedative–hypnotic agents, including benzodiazepines and atypical antipsychotics, are still frequently prescribed off‐label to elderly individuals with dementia, despite their inclusion among potentially inappropriate medications by the AGS Beers Criteria (American Geriatrics Society Beers Criteria 2023 Update). Recent analyses have shown that such prescriptions remain widespread in patients with insomnia and AD and are associated with increased incidence of adverse events, greater healthcare resource utilization, and higher overall treatment costs (Chekani et al. 2025).

In this context, such possible clinical application of lavender EO warrants further attention. Indeed, although the majority of clinical data are limited to subjective endpoints, the consistency of the findings strengthens the rationale for investigating lavender EO as a non‐invasive approach to modulate sleep–wake dynamics and restore oscillatory activity in prodromal AD. In general, further clinical studies, guided by quantitative EEG and focused on sleep–wake biomarkers, would clarify the neurophysiological relevance of lavender EO and its constituents in early AD. One such attempt to address this gap comes from a recent pilot study by Lee et al. (2023), which explored the effects of transdermal aromatherapy on sleep‐related biomarkers in institutionalized patients with dementia and comorbid insomnia. While several EOs were tested, lavender EO emerged as particularly effective in reducing physiological arousal: its application resulted in a significant decrease in 24‐h urinary free cortisol levels, suggesting a dampening of hypothalamic–pituitary–adrenal axis activity. This neuroendocrine effect was paralleled by behavioral improvements, including a reduction in daytime napping and nocturnal awakenings (Lee et al. 2023). Although limited by a small sample and the absence of blinding, the study offers preliminary biomarker‐based clinical evidence supporting the hypothesis that lavender EO may exert sleep‐promoting effects in neurodegenerative populations. These findings further justify the implementation of mechanistically oriented trials using objective physiological endpoints to clarify the contribution of lavender‐based interventions to sleep architecture and oscillatory homeostasis in early AD.

### 4.2 Lavender and Seizure Susceptibility: A Potential Link via Sleep and E/I Imbalance

Both the EO and hydroalcoholic extract of lavender have shown protective effects in a model of PTZ‐induced seizures, where they significantly increased seizure latency and survival percentage, although the exact mechanisms were not identified (Koutroumanidou et al. 2013; Rahimian et al. 2023). A recent preclinical study reported that a brain‐targeted formulation of lavender EO co‐delivered with apocynin via lactoferrin‐coated lipid nanocapsules significantly increased seizure latency, reduced seizure severity, and restored redox and inflammatory balance in a PTZ‐induced seizure model. Notably, this nanocarrier system enhanced brain bioavailability and promoted neuroprotection through both pharmacological and olfactory‐mediated mechanisms, suggesting a promising multimodal strategy for modulating neuronal excitability (Youssef et al. 2025). Beyond these studies, however, evidence supporting the anticonvulsant properties of lavender remains limited.

As previously discussed in relation to sleep regulation and cognition, lavender has attracted interest due to its capacity to modulate olfactory‐mediated mechanisms, including those related to the emerging link between the olfactory system and epileptogenesis. In particular, preliminary clinical data have reported beneficial effects of olfactory training involving lavender EO. A recent study by Yilmaz et al. (2022) showed that the inhalation of lavender aroma for 3 months in epileptic patients resistant to levetiracetam and carbamazepine induced a decrease in seizure frequency and duration, an increase in quality of life, and an improvement in olfactory function (Yilmaz et al. 2022). Notably, since the study was designed on olfactory training, the primary hypothesis focused on the established correlation between the olfactory system and epileptogenesis (Jiang et al. 2015; Yang et al. 2006). Nonetheless, the inhibition of seizure propagation could reflect the reported effects of linalool and lavender EO on the GABAergic system.

In light of these findings, and considering the correlation between brain rhythmicity, sleep‐related mechanisms, and network activity, the beneficial potential of lavender in relation to sleep disturbances and associated seizure susceptibility deserves further attention. Indeed, while the disruption of sleep and circadian rhythms is a main symptom of E/I imbalance in both humans and animal models of epilepsy (Matos et al. 2011; Samsonsen et al. 2016; Lanigar and Bandyopadhyay 2017), it has also been noted that restoring sleep can significantly improve the control of seizures (Lanigar and Bandyopadhyay 2017; Roundtree et al. 2016), hence suggesting that sleep disturbances may exacerbate E/I imbalance. In Tg2576 mice, a well‐known transgenic model of AD characterized by early hypersynchrony, altered sleep architecture was found to correlate with the occurrence of epileptiform activity events, likely due to abnormal pyramidal neuron activity and hippocampal excitability levels during REM sleep (Szabo et al. 2023). Importantly, theta oscillations, which can exert an antiepileptic role (Miller et al. 1994), were strongly disrupted by the epileptic events, a phenomenon that was correlated with the failure of inhibitory activity (Szabo et al. 2023). In addition, the elimination of theta oscillations during REM sleep has been shown to impair hippocampal‐dependent memory, consistent with their relevance in memory consolidation processes (Boyce et al. 2016).

In this context, it would be particularly important to explore in depth the relationship between sleep disturbances, epileptiform activity, and memory impairment in AD along with the potential strategies aimed at interfering with this pathogenic cycle. In this respect, studies examining in detail the neurobiological changes triggered by lavender inhalation or aromatherapy, particularly those underlying its effects on brain activity during sleep, would clarify whether lavender could potentially serve as a complementary approach to treat neuronal excitability disorders when used in combination with conventional drugs.

### 4.3 Lavender in AD: Experimental and Clinical Evidence

In the context of AD, both experimental and clinical studies have investigated the potential of lavender EO, with preclinical work focusing on cognitive performance and neuroprotection, and clinical trials primarily addressing behavioral symptoms and quality of life. In particular, lavender EO displayed antioxidant and antiapoptotic effects, and the ability to protect against Aβ_1‐42_ injury in vitro (Caputo et al. 2021). In agreement, lavender EO was able to improve cognitive performance in mice with scopolamine‐induced dementia in behavioral tests, and to protect cells from oxidative stress (Xu et al. 2016). In a rat model of scopolamine‐induced dementia, lavender EO was able to improve spatial memory in the radial arm‐maze task, suggesting its ability to sustain the hippocampus‐dependent memory formation (Hritcu et al. 2012). In the same experimental model, lavender EO significantly diminished the anxiety‐like behavior in the elevated plus maze task, in line with the numerous studies reporting on the anxiolytic‐like effect of lavender in both animals and human patients (Chioca et al. 2013; Woelk and Schläfke 2010; von Känel et al. 2021).

In fact, lavender EO inhalation showed to be effective in numerous trials involving dementia patients, mostly in managing some psychobehavioral alterations such as agitation and anxiety (Lin et al. 2007; Fujii et al. 2008; van der Ploeg et al. 2010; Kimura and Takamatsu 2013; Watson et al. 2019). A randomized controlled trial by Watson et al. further confirmed the feasibility and tolerability of lavender EO aromatherapy for reducing agitation in elderly individuals with and without dementia, although the intervention combined lavender with lemon balm and did not assess cognitive outcomes directly (Watson et al. 2019). Similarly, a recent RCT conducted by Li et al. (2025) tested a home‐based aromatherapy protocol administered by trained caregivers and found significant reductions in disinhibition and irritability in dementia patients after 3 weeks of lavender EO inhalation, along with improvements in quality‐of‐life domains (Li et al. 2025).

These findings are consistent with a meta‐analysis of 15 RCTs that evaluated the impact of aromatherapy on agitation and aggression in cognitively impaired populations, showing that inhalation of lavender EO was the most effective modality in reducing behavioral disturbances, especially when delivered in short‐term interventions (≤ 4 weeks) (Xiao et al. 2021). Although cognitive outcomes were rarely assessed in these trials, the convergence of behavioral, emotional, and sleep‐related effects strengthens the rationale for the therapeutic relevance of lavender in AD care. Notably, one study testing a multicomponent aromatherapy intervention on AD patients reported improvements in behavioral functioning that indirectly supports cognitive engagement, although the specific contribution of lavender could not be isolated (Jimbo et al. 2009).

Overall, the anxiolytic and sedative properties of lavender, its capacity to improve sleep quality, and its favorable effects on behavioral symptoms may converge in modulating neuronal network activity and reducing cognitive vulnerability in AD. Nonetheless, further large‐scale clinical trials are warranted to confirm efficacy, optimize standardization, and elucidate mechanisms of action underlying lavender's potential as a network‐level intervention in AD.

## 5 Cannabis

*Cannabis sativa* L., generically known as cannabis, is a flowering herbaceous plant that has been used by humans for thousands of years for both medicinal and recreational purposes. Although all the parts of the cannabis plant were used in traditional medicine, the inflorescences are currently the most used part in the pharmaceutical industry. At present, the therapeutic potential of cannabis is widely recognized, and the number of medicinal cannabis users is increasing rapidly. Cannabis is indeed prescribed for different medical conditions, including chronic and cancer‐related pain, chemotherapy‐associated nausea and vomiting, Tourette's syndrome, and refractory pediatric epilepsy when first‐line treatments do not produce the desired results. Cannabis has also shown potential benefits in alleviating symptoms of depression, anxiety, autism, and neurodegenerative diseases (Bitencourt et al. 2021). However, the lack of standardized formulations in terms of bioactive components, especially for the galenic preparations, prevents full clinical exploitation of its therapeutic potential. This lack of standardization stems from multiple factors, including the genetic diversity across cannabis cultivars (Mandolino et al. 2003), the impact of cultivation conditions such as light, nutrient availability, and planting density on phytochemical expression (Danziger and Bernstein 2022, 2021), and the uneven distribution of bioactive compounds across different plant organs. Together, these factors result in substantial batch‐to‐batch variability in the content of cannabinoids and minor constituents such as terpenes and flavonoids, thereby complicating both the interpretation of experimental results and the reproducibility of therapeutic responses in clinical settings.

Beyond chemical composition, another key issue affecting clinical reproducibility is the mode of administration. Although medicinal cannabis is administered by smoking in most cases, this route of administration is accompanied by well‐known health‐threatening issues such as the inhalation of ash particles and toxic oxidation products and suffers from poor reproducibility. Likely, a preferred delivery form is based on the cannabis oils obtained from inflorescences. These cannabis extracts, which can be administered sublingually, via oral spray, or as capsules, permit safer use and provide higher accuracy and reproducibility. Nonetheless, significant differences in the composition of the consumed oils have been found in comparison with that of the corresponding smoked inflorescences. This evidence has prompted researchers to focus more closely on characterizing the amount and the pharmacological impact of each class of components in cannabis extracts for therapeutic use. In addition to phytocannabinoids, which are exclusively produced in cannabis and have been identified as the most relevant compounds for their broad poly‐pharmacology, the cannabis plant also synthesizes a variety of molecules such as terpenoids and flavonoids that could participate in its biological activity hence determining the so‐called “entourage effect” (Ben‐Shabat et al. 1998). The phytocannabinoid class includes more than a hundred compounds (ElSohly et al. 2017), although the most pharmacologically relevant are CBD and Δ^9^‐tetrahydrocannabinol (Δ^9^‐THC), the latter known as the main psychoactive component of cannabis. The terpenoid class is even more numerous, comprising more than 200 different terpenes. The most prevalent terpenes include myrcene, terpinolene, limonene, α‐pinene, humulene, linalool, and β‐caryophyllene (Fischedick 2017), with myrcene levels providing the strongest demarcation between the *sativa*‐type and *indica*‐type *Cannabis*. Linalool, in particular, may represent a significant portion (up to 6%) of cannabis extract composition (Russo and Marcu 2017). In general, the relative proportions of these compounds may differ significantly between cannabis‐derived products, depending on their extraction method and botanical source, posing further challenges to the reproducibility and predictability of their pharmacological effects.

Both CBD and Δ^9^‐THC exert a broad spectrum of pharmacological activities by interacting with multiple molecular targets. However, the primary pharmacological effects of Δ^9^‐THC and CBD are mediated by the interaction with the endocannabinoid system and with the cannabinoid receptors 1 and 2 (CB1 and CB2), in particular. CB1 and CB2 receptors exert a wide range of effects on the nervous system by regulating multiple functions, such as neuronal homeostasis, inflammation, and neurotransmission (Sugaya and Kano 2022). The activation of CB1 receptors located presynaptically inhibits the activation of voltage‐gated Ca^2+^ channels and increases K^+^ channel activity, hence inducing a transient hyperpolarization and inhibiting the release of neurotransmitters like glutamate and GABA (Shen et al. 1996; Wilson et al. 2001). Importantly, CB1 receptors are widely expressed in the hippocampus, where they have been linked to network excitability and synaptic plasticity regulation, and therefore to learning and memory functions (Durieux et al. 2022; Winters and Vaughan 2021). CB2 activation primarily leads to immunosuppressive responses in immune cells or organs (Galiègue et al. 1995), even if a limited expression of CB2 receptors has been found in specific regions of the CNS such as the hippocampus and brain stem, where their activation may induce neuronal excitation (Stempel et al. 2016). CBD is thought to mainly act as a negative allosteric modulator of the cannabinoid receptor CB1 and as an antagonist/inverse agonist of CB2, whereas Δ^9^‐THC acts as a partial agonist on both CB1 and CB2 (Laprairie et al. 2015; Shen and Thayer 1999).

### 5.1 Phytocannabinoids in AD: Neuroprotective Effects and Interaction With Disease Mechanisms

CB1 and CB2 receptors, along with endocannabinoids and the enzymes involved in their synthesis or degradation, have been found to be altered in AD. Their expression appears to be up‐regulated in the hippocampus and cerebral cortex during the early stages of the disease and to decrease in the later phases (Farkas et al. 2012; Manuel et al. 2014). Although the exact pathological significance of these alterations remains unclear, the endocannabinoid system has emerged as a potential target for novel therapeutic strategies in AD.

Pre‐clinical investigations have shown that both phytocannabinoids and endocannabinoids may exert beneficial effects on dementia‐related symptoms. Both CBD and Δ^9^‐THC have displayed therapeutic potential by modulating several mechanisms implicated in the AD pathogenesis. In addition to its antioxidant effect observed in vitro (Coles et al. 2022), CBD has exhibited anti‐inflammatory and cognitive‐enhancing properties in in vivo experimental models of AD (Martín‐Moreno et al. 2011; Kreilaus et al. 2022). Interestingly, recent medicinal chemistry studies have explored structurally modified CBD derivatives as potential anti‐AD agents. Notably, certain CBD–carbamate hybrids have demonstrated selective butyrylcholinesterase inhibition and reduced brain Aβ levels in transgenic mouse models (Jiang et al. 2021), while pharmacokinetic profiling has revealed favorable brain‐targeting properties for specific compounds (Wang et al. 2024).

Regarding Δ^9^‐THC, it was found to inhibit Aβ aggregation and to protect cells from oxidative stress and excitotoxicity in in vitro experiments (Schubert et al. 2019), while several in vivo studies have reported its ability to promote neurogenesis and reverse age‐related cognitive impairment in aged mice (Prenderville et al. 2015; Sarne et al. 2018; Bilkei‐Gorzo et al. 2017; Nidadavolu et al. 2021). Notably, the long‐term neuroprotective effects of ultra‐low doses of Δ^9^‐THC (0.002 mg/kg) have also been demonstrated in aged mice (Fishbein‐Kaminietsky et al. 2014; Assaf et al. 2011). In general, existing data suggest that low doses of Δ^9^‐THC may be well tolerated in dementia patients (van den Elsen, Ahmed, Verkes, Kramers, et al. 2015; van den Elsen, Ahmed, Verkes, Feuth, et al. 2015), although no significant clinical benefits have been clearly demonstrated to date.

Different outcomes have been reported in AD experimental models when Δ^9^‐THC was combined with CBD at varying ratios. In particular, Δ^9^‐THC‐rich, CBD‐rich, or 1:1 CBD:Δ^9^‐THC extracts reversed memory deficits in transgenic AD mice (Aso et al. 2015), suggesting that multi‐cannabinoid formulations may represent a promising therapeutic approach to be further explored in this context. In fact, it is well established that phytocannabinoids can interact synergistically or antagonistically when administered in combination, influencing the overall therapeutic profile of the preparation (Russo and Guy 2006). This so‐called “entourage effect” (see Section 5.3) is particularly relevant when considering the potentially detrimental effects of Δ^9^‐THC, since CBD—and likely other phytocannabinoids—may attenuate its negative effects on cognition and memory (Straiker et al. 2018; Hudson et al. 2019; Englund et al. 2013), while enhancing its beneficial properties (Hayakawa et al. 2008).

### 5.2 Modulation of Neuronal Excitability by Phytocannabinoids: Implications for Seizure Activity and E/I Balance

A growing body of preclinical and clinical evidence supports the anticonvulsant activity of cannabis. Phytocannabinoids, especially Δ^9^‐THC and CBD, have displayed anticonvulsant effects in various experimental seizure models (Rosenberg et al. 2017). As a result, the number of clinical studies evaluating their therapeutic use for refractory epilepsy has increased significantly in recent years. However, due to the complex psychoactive and somatic side effects of Δ^9^‐THC, most clinical investigations have focused on CBD. Nonetheless, in most clinical studies, CBD has not been administered as an isolated compound but as part of complex cannabis extracts with high CBD:Δ^9^‐THC ratios. This is particularly relevant given that such extracts have shown greater efficacy and tolerability compared with purified CBD (Pamplona et al. 2019), suggesting a possible contribution of other constituents, including terpenes, to the overall therapeutic effect. Overall, these studies have provided solid demonstrations that cannabis is effective in treating various forms of drug‐resistant epilepsy and displays a favorable side‐effect profile (Rubin 2018; Metternich et al. 2021). As a result, a cannabis‐derived medication containing > 98% CBD, along with other cannabinoid and non‐cannabinoid compounds, was approved by the Food and Drug Administration in 2018 and by the European Medicines Agency in 2019 as an add‐on treatment for childhood refractory epilepsies (Raucci et al. 2020).

It is worth noting that, while Δ^9^‐THC primarily exerts its effects via CB1 receptor activation, as extensively reported in the literature, the mechanisms underlying the anticonvulsant activity of CBD are more complex and still not fully understood. Several CNS proteins, particularly at the hippocampal level, have been identified as CBD targets in preclinical studies, including several ion channels. These CB1‐independent pathways may help explain why CBD displays anticonvulsant effects despite acting as a CB1 antagonist (Devinsky et al. 2014). Some data have demonstrated the ability of CBD to modulate Na_V_ channels. Ghovanloo et al. (2018) showed that CBD inhibited Na_V_ currents with an IC_50_ of 1.9–3.8 μM by preventing channel opening and stabilizing the inactivated state of the channels (Ghovanloo et al. 2018). Ross et al. (2008) found that both CBD and Δ^9^‐THC inhibited the voltage‐gated calcium (Ca_V_) channels Ca_V_3.1 and Ca_V_3.2 with an IC_50_ of ∼1 μM, and Ca_V_3.3 with less potency, producing a significant hyperpolarizing shift in the steady‐state inactivation of these channels (Ross et al. 2008). Notably, CBD also acts as a positive allosteric modulator of GABA_A_ receptors (Bakas et al. 2017). In line with this, the intraperitoneal administration of CBD (60 mg/kg) reduced tonic seizures induced by PTZ in rats (Consroe et al. 1982).

Although the anticonvulsant effects of CBD do not appear to be primarily mediated by cannabinoid receptors, CB1 receptor activation and overexpression have been implicated in the reduction of seizure severity and neuronal cell death (Monory et al. 2006; Guggenhuber et al. 2010; Huizenga et al. 2017). In parallel, endocannabinoids have been reported as neuroprotective in models of acute seizure activity (Wallace et al. 2002). Δ^9^‐THC has also been shown to decrease excitatory neurotransmission, reduce hippocampal overexcitation, and attenuate seizure severity in various murine models (Shen and Thayer 1999; Wallace et al. 2003; Corcoran et al. 1973), confirming that the CB1 receptor can serve as a pharmacological target in hyperexcitability disorders.

### 5.3 Terpenes‐Phytocannabinoids Entourage Effect: Potential Pharmacological Relevance of Linalool in Cannabis Extracts

The “entourage effect” refers to a pharmacodynamic synergy in which multiple cannabis‐derived compounds, both active and those previously considered inactive, interact to modulate and enhance therapeutic outcomes (Ben‐Shabat et al. 1998). Originally introduced in the context of endocannabinoid signaling, the concept has since been extended to include phytocannabinoids and terpenes (Russo 2011). Synergistic mechanisms may involve multi‐target modulation, improved bioavailability, attenuation of adverse effects, or amplification of primary pharmacological actions. Experimental evidence has shown that whole‐plant cannabis extracts often exert greater effects than isolated Δ^9^‐THC or CBD, supporting the idea that minor cannabinoids and terpenes, such as linalool, may significantly influence the efficacy and tolerability of cannabinoid‐based therapies.

This notion was reinforced by early observations that smoked cannabis inflorescences often produced different clinical and behavioral responses compared with purified compounds or standardized extracts. Such discrepancies, partly attributed to the loss of volatile constituents during processing, led to the hypothesis that minor components, particularly terpenes, might contribute to the overall pharmacological effect (Russo 2011).

Notably, linalool has been shown to induce a 73% reduction in motility at 4.22 ng/mL, a concentration comparable to the Δ^9^‐THC levels associated with therapeutic efficacy in randomized controlled trials for pain and multiple sclerosis (Russo 2008). Case reports have also suggested that linalool‐rich formulations may enhance seizure control or behavioral outcomes when compared with similar preparations with lower or absent terpene content (Sulak et al. 2017; Raz et al. 2022), reinforcing the hypothesis that the anti‐convulsant properties of full‐spectrum cannabis extracts may involve linalool and/or other terpenic components acting through complementary mechanisms.

Consistent with this view, a recent study reported synergistic anxiolytic effects to be exerted by linalool and CBD at sub‐effective levels, suggesting that such a combination could be harnessed to expand the effective dose range of the primary phytocannabinoid (Wagner et al. 2024). Another preclinical study by Degrave et al. demonstrated that cannabis oils with different CBD:THC ratios and defined terpene profiles, including linalool, produced beneficial effects on hepatic inflammation, oxidative stress, and CB1 receptor expression in a rat model of metabolic syndrome (Degrave et al. 2025). Additional support comes from a recent double‐blind, placebo‐controlled crossover trial in patients with severe insomnia, where a CBD formulation enriched with eight individual terpenes, including linalool, significantly increased the proportion of restorative sleep, defined as the combined duration of slow‐wave and REM sleep stages. The effect, which was not associated with sedation or adverse events, was particularly pronounced in individuals with poor baseline sleep quality or altered circadian rhythms (Wang et al. 2025).

Although the molecular basis of cannabinoid–terpenoid interactions remains to be fully clarified, recent evidence indicates that certain terpenes, including linalool, may modulate CB1 receptors through low‐affinity binding or indirect mechanisms (Santiago et al. 2019; Donatello et al. 2020; Moore et al. 2023) (see Section 3.4). As discussed in Section 3.4, linalool has also been shown to modulate A2A receptors and to produce antinociceptive effects in animal models independently of CB1 activation. This suggests that its pharmacological activity may rely on broader neuromodulatory pathways, with potential implications for seizure control and neuroinflammation attenuation by cannabis extracts.

Altogether, these findings suggest that terpene composition may significantly influence the efficacy of cannabinoid‐based formulations beyond cannabinoid content alone. Specific cannabinoid–terpene combinations, such as those including linalool, may therefore enhance the therapeutic index of cannabis extracts, particularly in epilepsy and AD. Since CB1 activation has been associated with seizure reduction and neuroprotection, the ability of linalool to modulate CB1 (and A2A) signaling could be relevant for achieving synergistic effects and improving clinical outcomes.

## 6 Translational Challenges and Future Perspectives

Despite the growing body of preclinical evidence supporting the neuroprotective and network‐modulating effects of linalool, clinical studies remain limited, especially in the context of AD. Most of the available trials have focused on behavioral or subjective outcomes such as anxiety and sleep quality, with limited attention to mechanistic endpoints—such as oscillatory dynamics, neuronal excitability, or disease‐related biomarkers—more directly related to E/I balance and network function.

In this regard, mechanistically oriented clinical studies would represent a necessary step forward. Approaches guided by quantitative EEG, polysomnography, or fluid biomarkers of neurodegeneration and network dysfunction could help clarify the translational potential of linalool‐containing formulations, particularly in early or prodromal AD, where neuronal hyperexcitability and altered sleep architecture are more prominent and potentially reversible. Moreover, given the increasing evidence of sex‐dependent differences in response to olfactory or terpene‐based interventions, sex‐stratified analyses and pharmacokinetic assessments should be systematically integrated into future study designs.

Standardization of the phytochemical composition is another critical aspect. In this context, important differences emerge between lavender and cannabis. Cannabis‐based preparations enriched with specific monoterpenes or other minor constituents are still challenged by the lack of phytochemical standardization. The chemical profile of *Cannabis sativa* L., including cannabinoids, terpenes, and other bioactive metabolites, can vary considerably depending on genotype, environmental conditions, and intra‐plant differences (Mandolino et al. 2003; Danziger and Bernstein 2021). This variability introduces significant challenges for reproducibility and complicates the comparison of results across studies, highlighting the need for more controlled approaches in both experimental design and product formulation. Conversely, the use of lavender is supported by a long tradition of clinical and preclinical research, particularly in the management of psychiatric and behavioral symptoms associated with AD. In addition, the composition of its EO is regulated by ISO standards and pharmacopoeial monographs, offering a higher degree of chemical consistency that facilitates experimental reproducibility and translational application.

Finally, combined interventions integrating linalool with established or experimental treatments could represent a promising avenue to explore potential synergistic effects, particularly in relation to sleep disturbances and subclinical epileptiform activity, which are increasingly recognized as early features of AD and potential targets for intervention.

## 7 Conclusions

Altogether, the available evidence supports the hypothesis that linalool may represent a promising candidate for modulating network dysfunctions in AD. Through its multimodal actions, linalool appears to target several processes critically involved in the E/I imbalance observed in the early stages of the disease. Although current findings are largely based on preclinical data, the convergence of its neuromodulatory, antioxidant, and behavioral effects provides a rationale for future translational efforts. The presence of linalool in clinically used botanical preparations, particularly lavender EO and cannabis extracts, further suggests potential applicability in human settings. Inhalation of lavender EO has already shown beneficial effects on anxiety, agitation, and sleep disturbances in dementia, while cannabis‐based formulations enriched in monoterpenes are under investigation for seizure control and cognitive support. These properties, together with a favorable safety profile, indicate that linalool‐containing interventions may offer additional therapeutic value when integrated within broader multimodal strategies targeting network dysfunction in AD. However, well‐designed clinical studies are needed to validate these hypotheses and clarify the relevance of linalool‐based approaches in the context of neurodegenerative disease.

## Author Contributions

**Anna Pannaccione:** writing – review and editing, supervision, conceptualization. **Ilaria Piccialli:** writing – original draft, writing – review and editing, conceptualization. **Giovanni Roviello:** writing – original draft, visualization, investigation. **Giorgia Magliocca:** writing – original draft, visualization, investigation. **Emilia Esposito:** writing – original draft, visualization, investigation. All authors have read and agreed to the submitted version of the manuscript.

## Funding

This work was supported by the Italian Ministry of University and Research (MUR), under the PRIN 2022 program (Grant No. 2022ZTKFXS); by MUR under the PNRR program, Project MNESYS (PE0000006).

## Conflicts of Interest

The authors declare no conflicts of interest.

## Data Availability

Data sharing not applicable to this article as no datasets were generated or analysed during the current study.
